# Supplementary material for: Structural basis for FGF hormone signalling
Source: Nature. 2023 Jun 7;618(7966):862–70. doi: 10.1038/s41586-023-06155-9 (PMC10284700; doi:10.1038/s41586-023-06155-9)
Supplement: Supplementary file 1 — Supplementary Figs. 1–4 and Tables 1–2. [file 41586_2023_6155_MOESM1_ESM.pdf]

nature portfolio

https://doi.org/10.1038/s41586-023-06155-9

Supplementary information

Structural basis for FGF hormone signalling

In the format provided by the authors and unedited

Supplementary Fig.1. Raw uncropped western blot images

Uncropped blotting for Figure 2

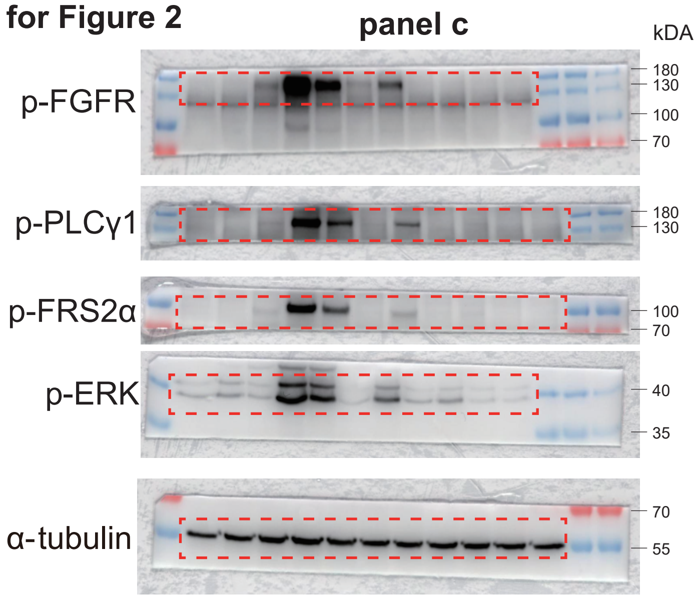

Uncropped blotting for Figure 3

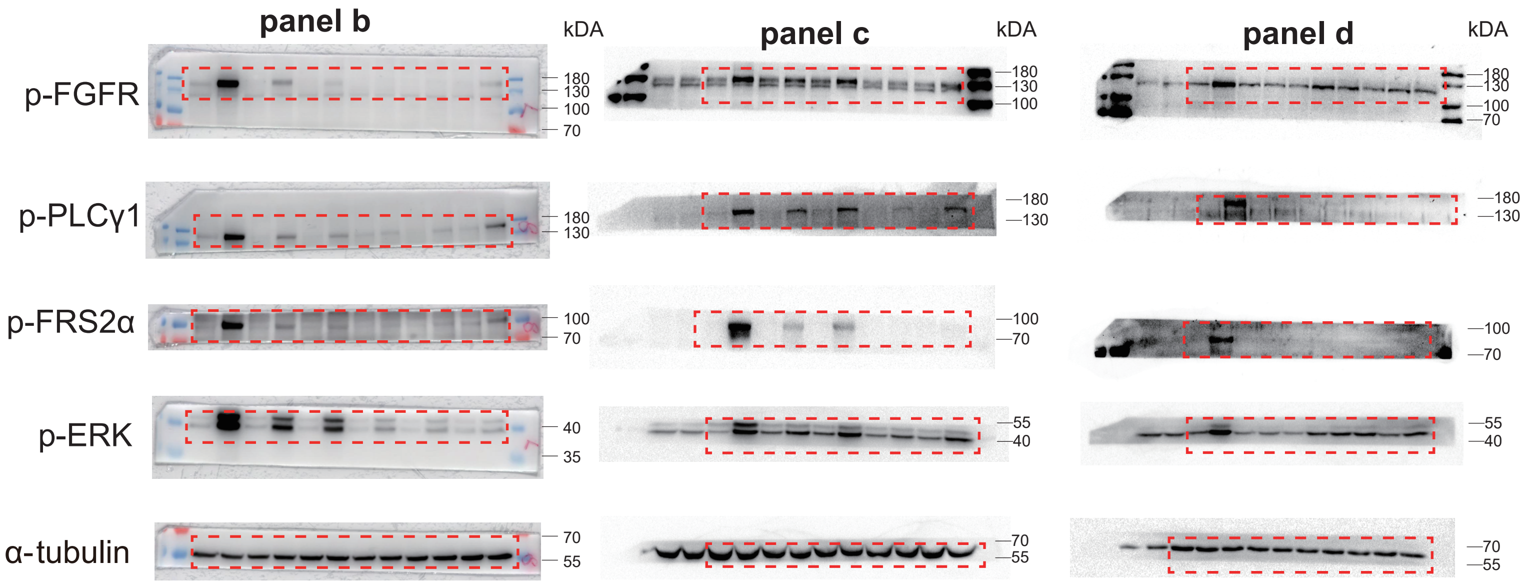

Uncropped blotting for Figure 4

panel b

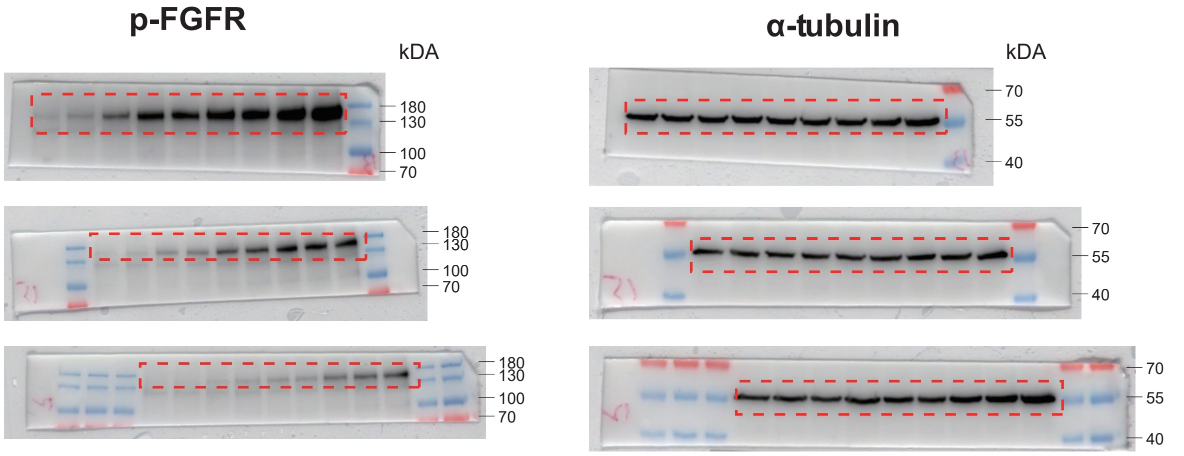

panel d

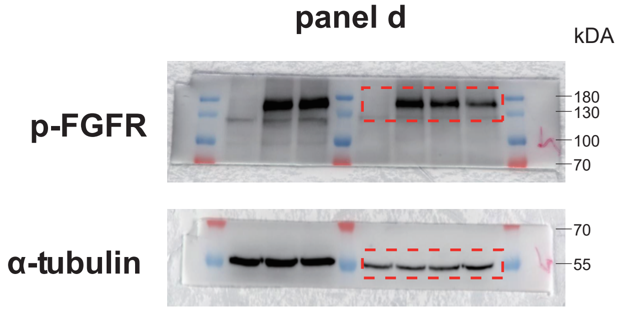

Uncropped blotting for Figure 5

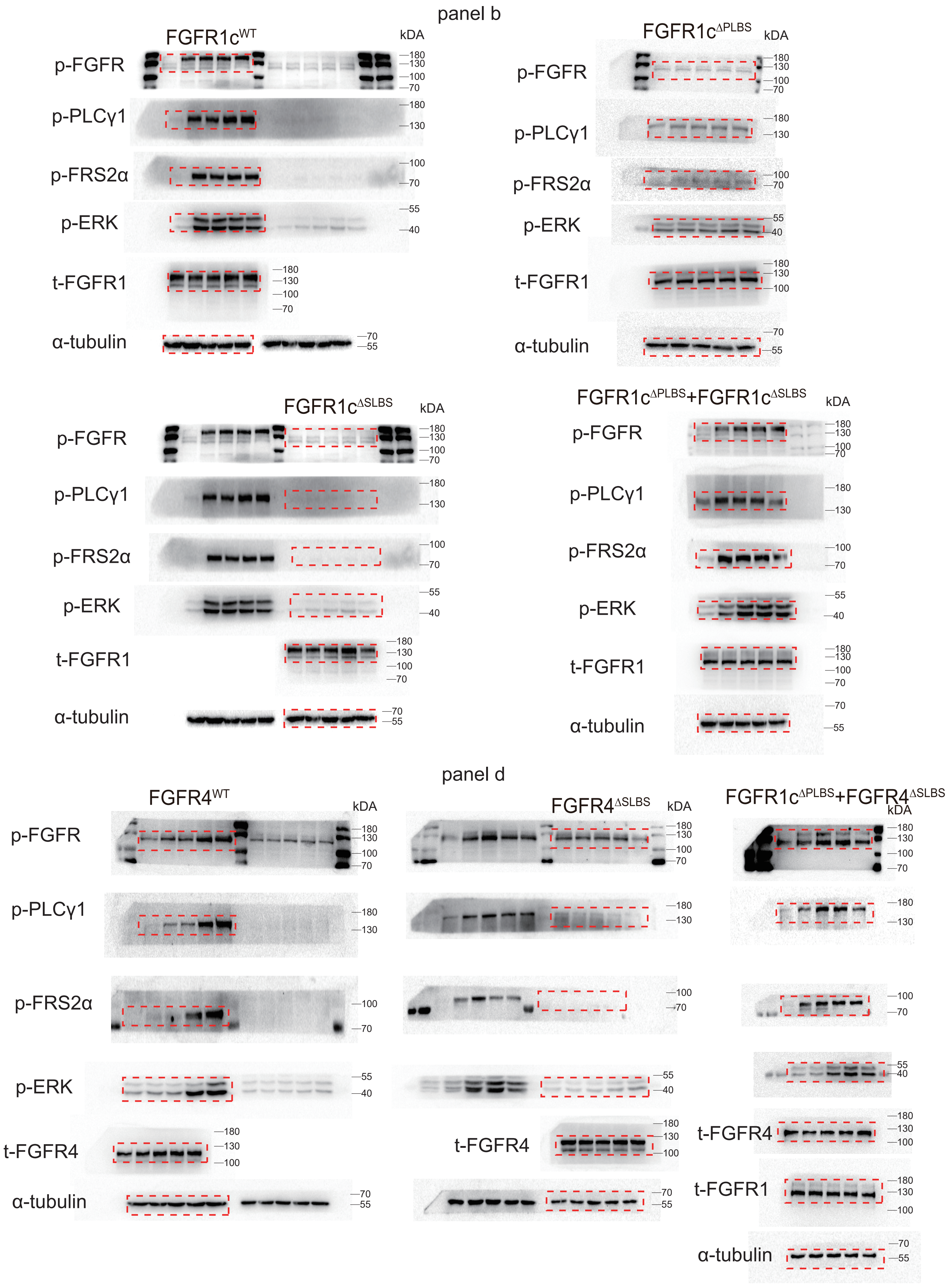

Uncropped Blotting for Figure 5

panel f for FGF1

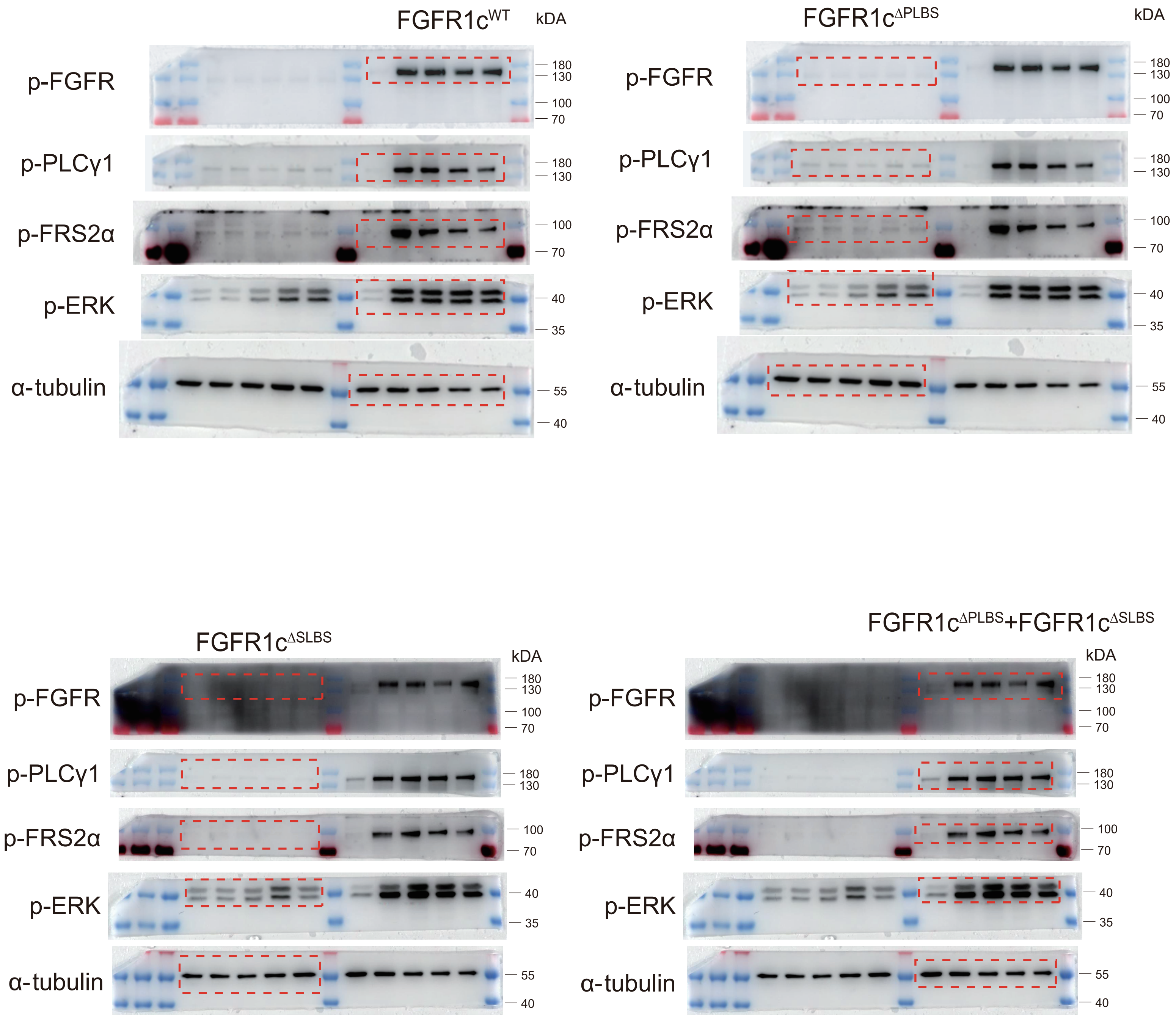

Uncropped Blotting for Figure 5

panel f for FGF4

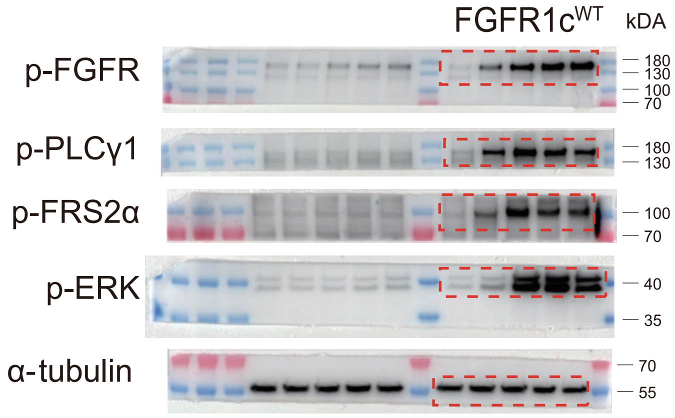

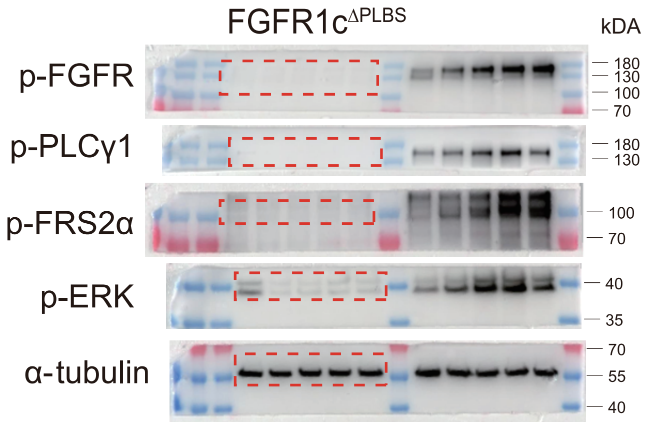

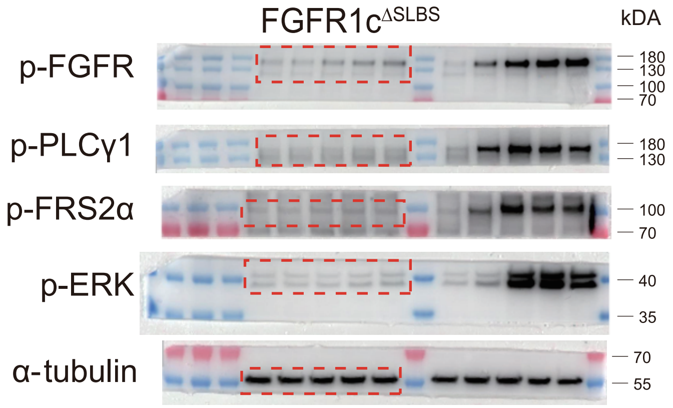

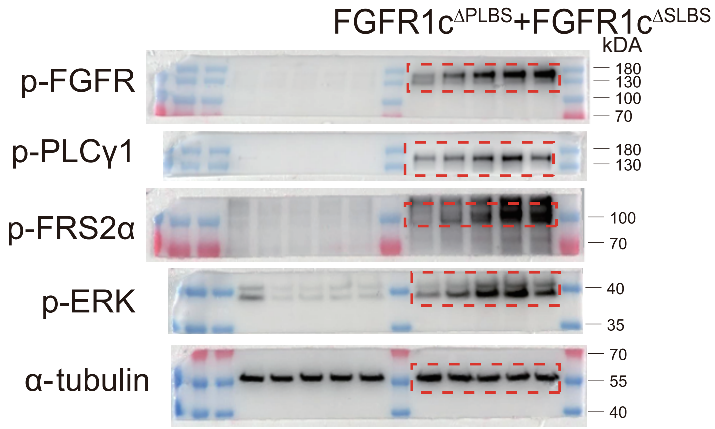

Uncropped Blotting for Figure 5

panel h

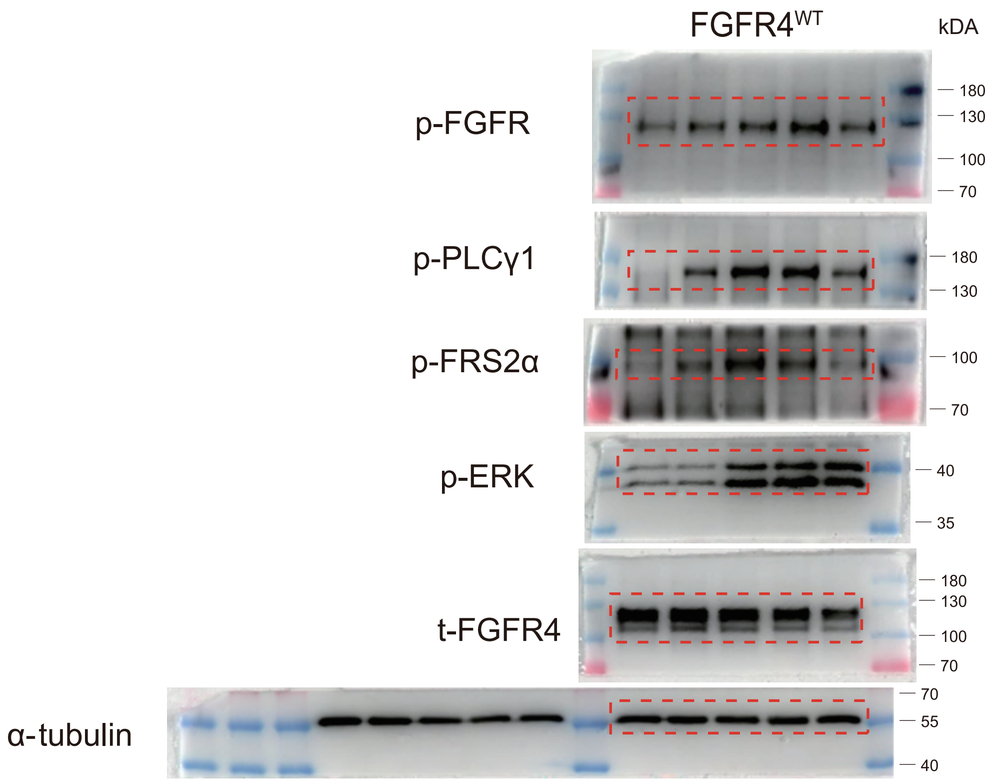

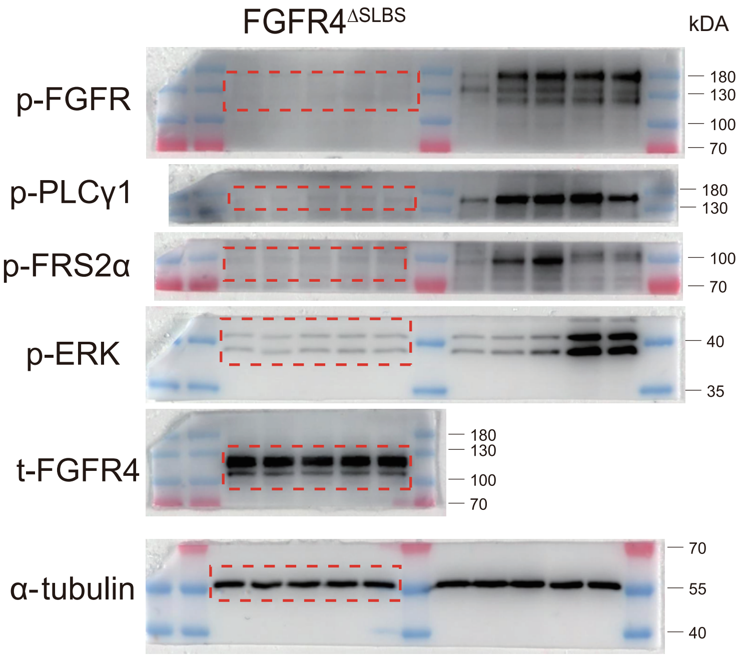

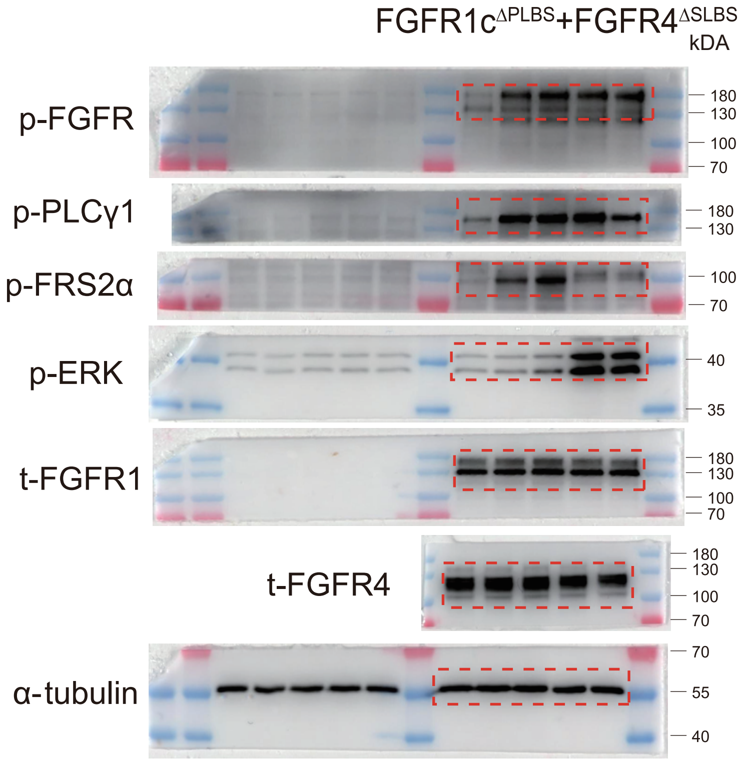

Uncropped blotting for Extended Data Figure 2

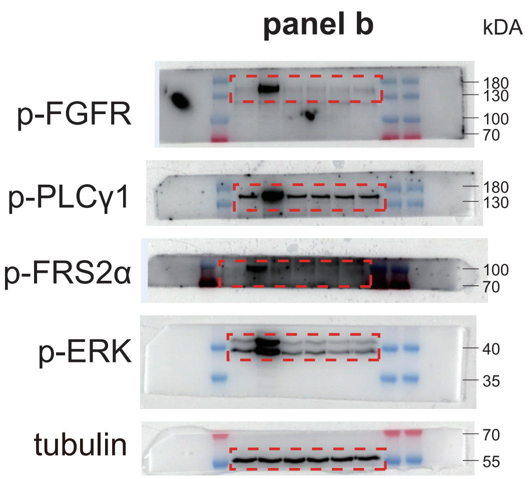

Uncropped blotting for Extended Data Figure 3

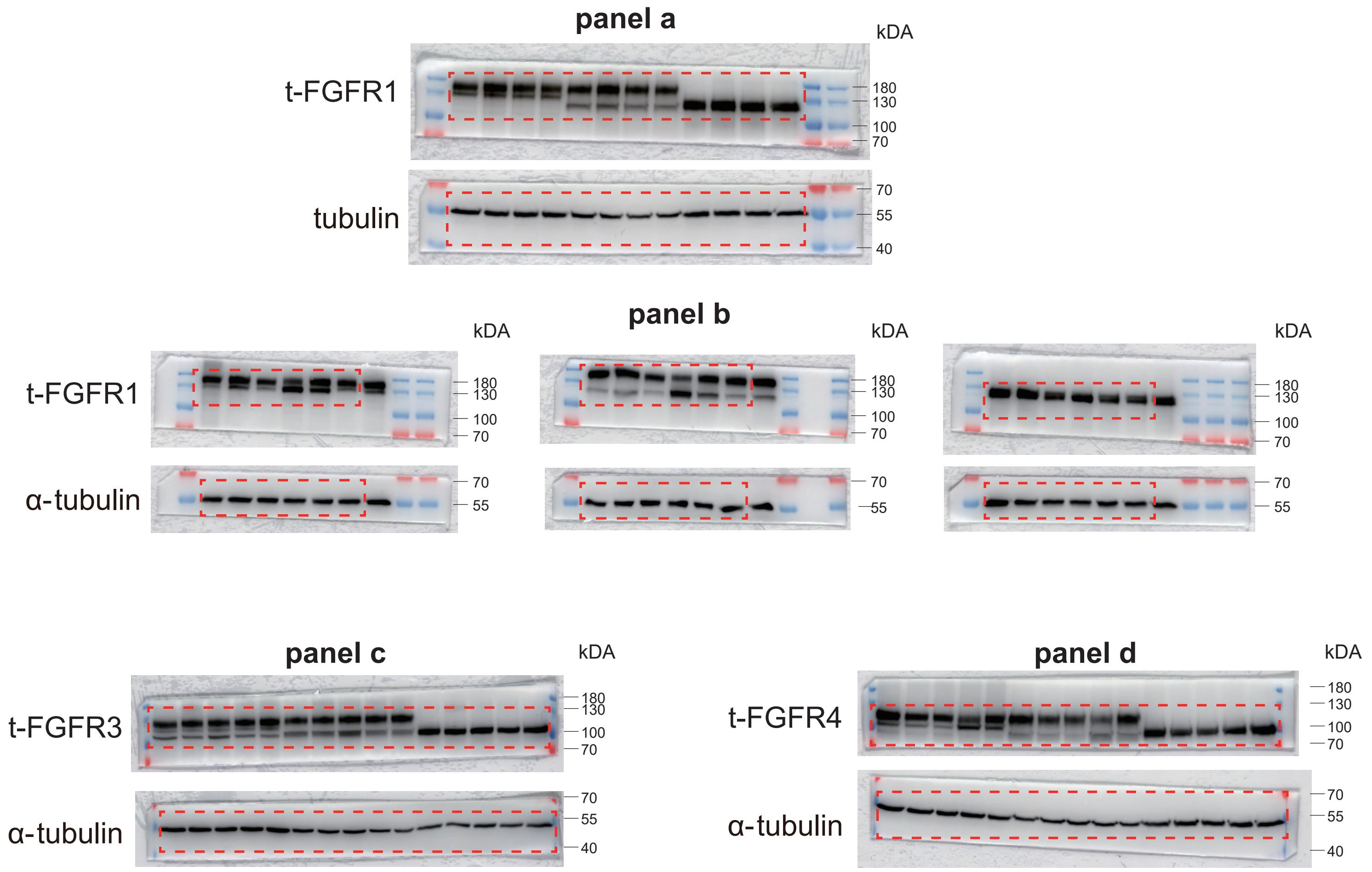

Uncropped Blotting for Extended Data Figure 6a

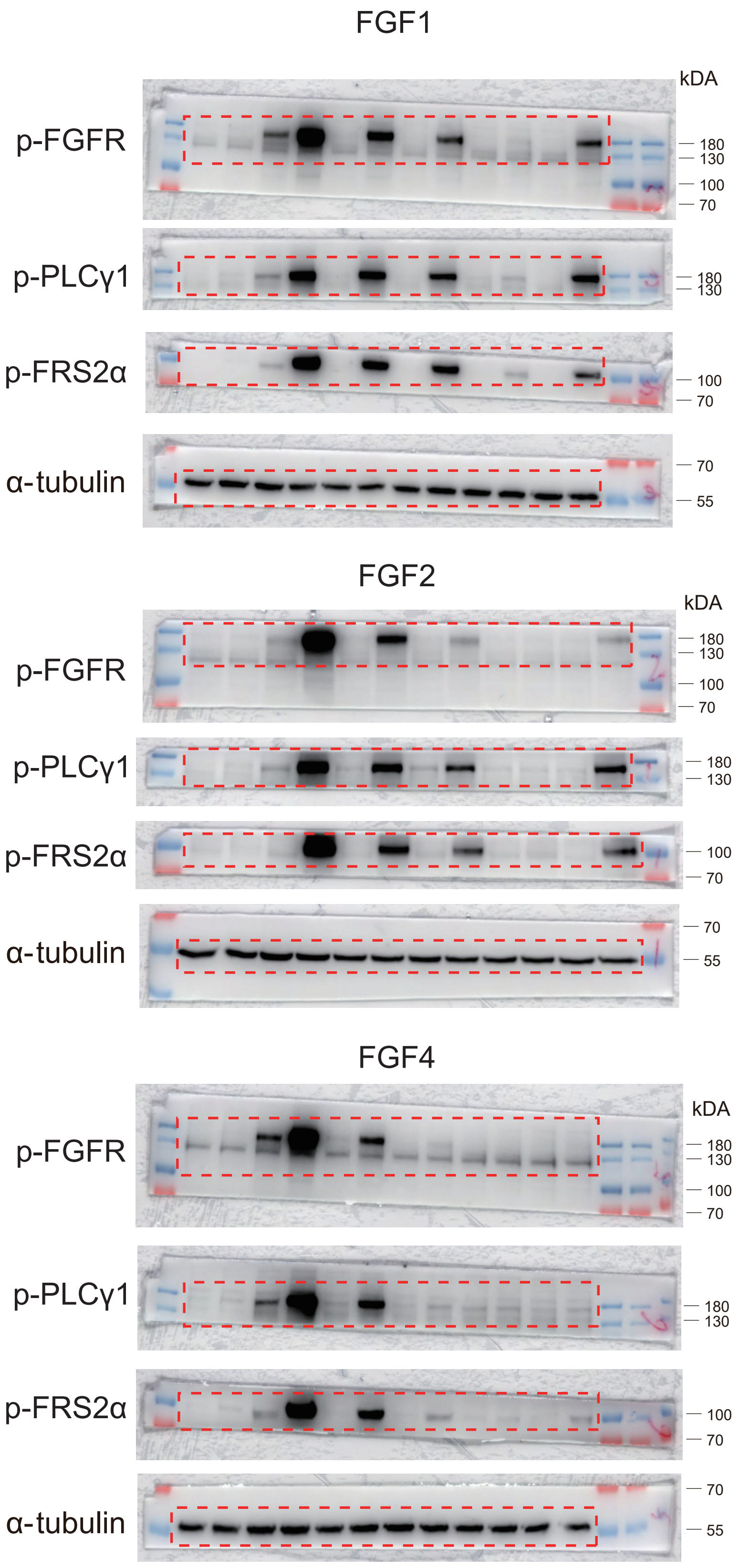

Uncropped Blotting for Extended Data Figure 6b

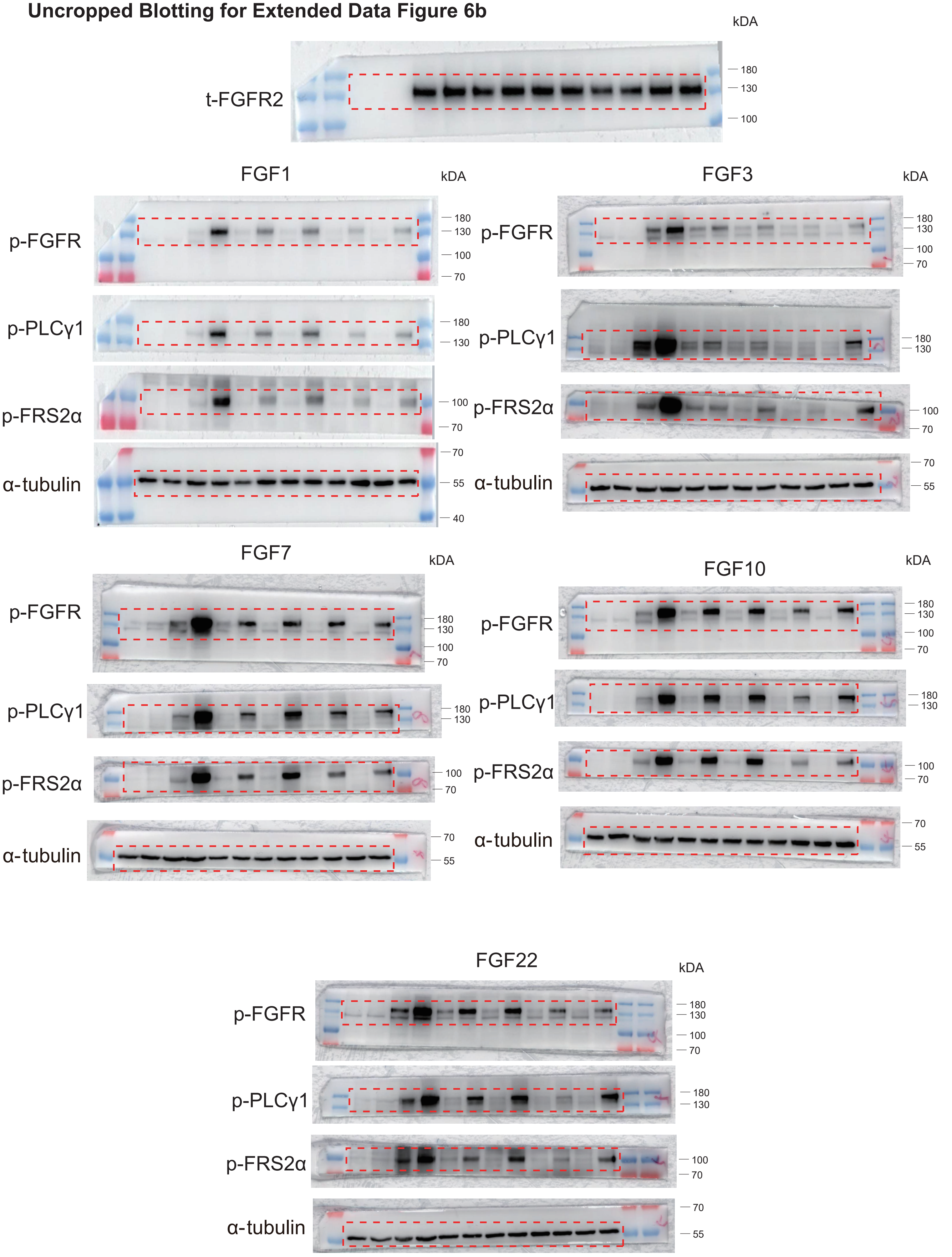

Uncropped Blotting For Extended Data Figure 7b

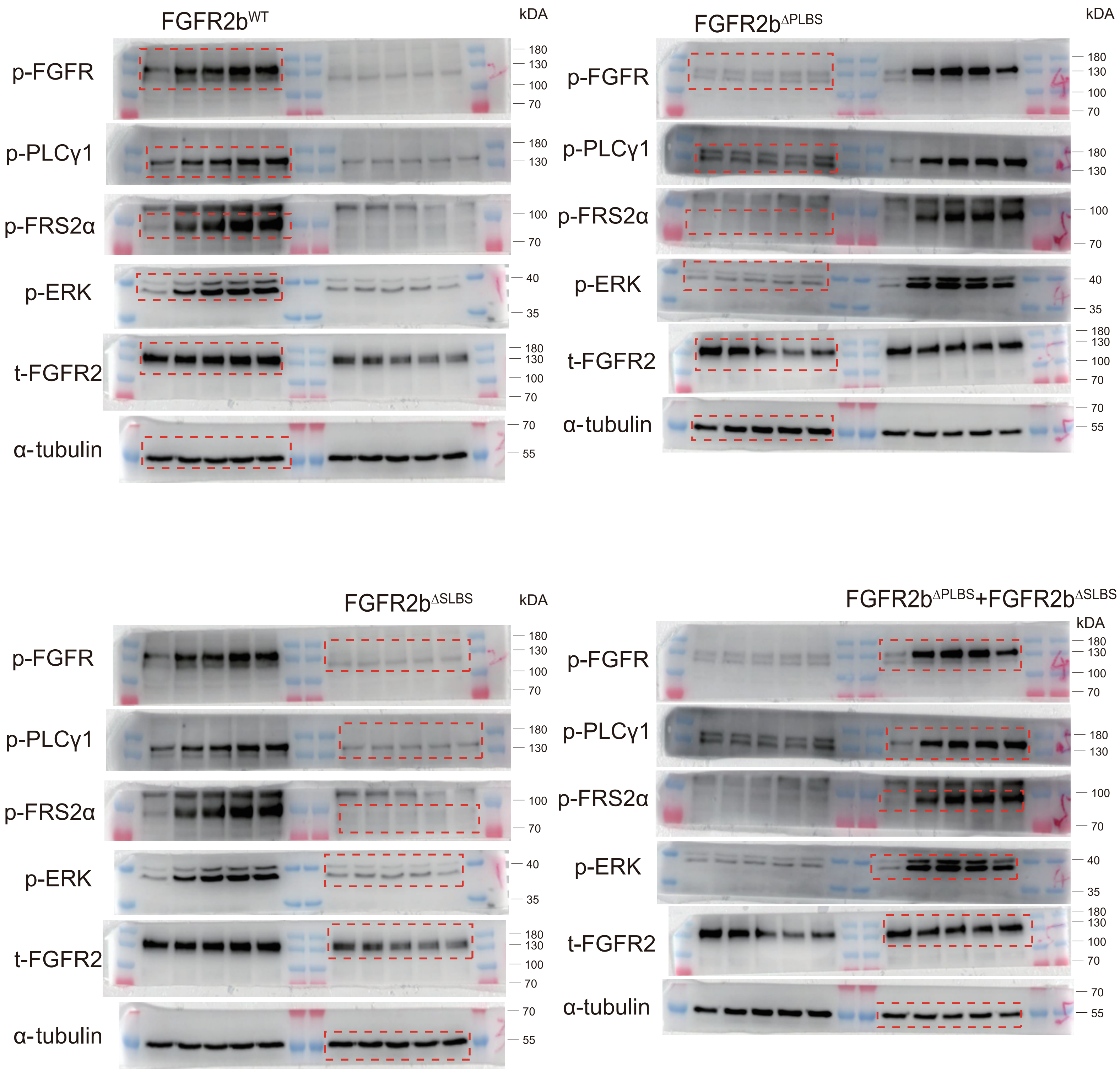

Uncropped Blotting For Extended Data Figure 7c

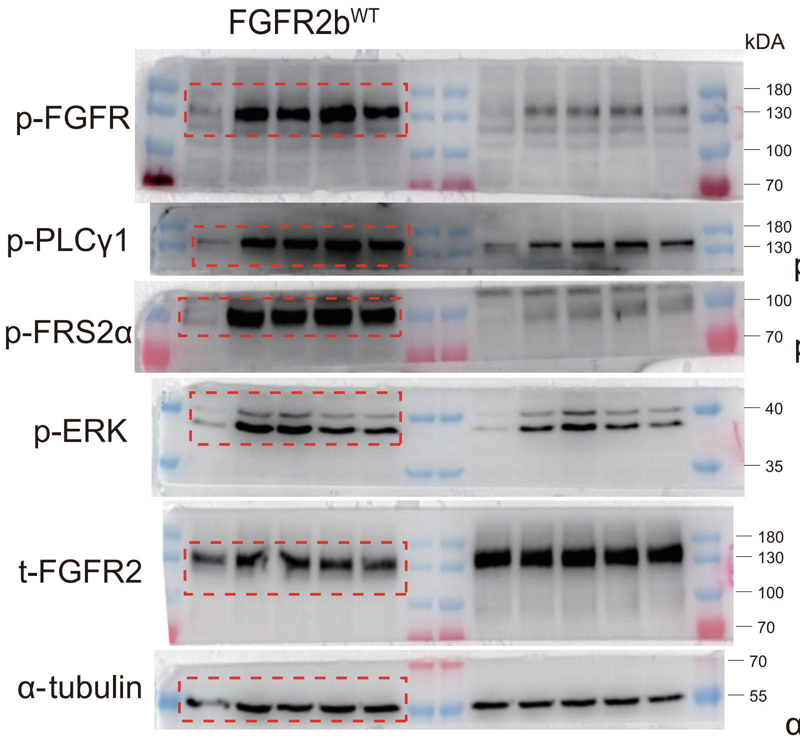

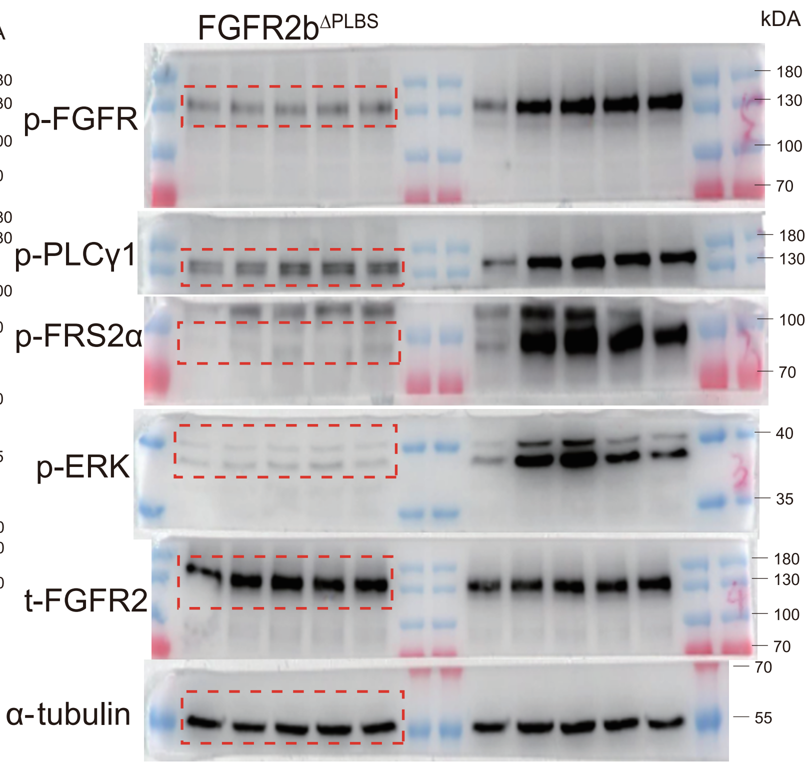

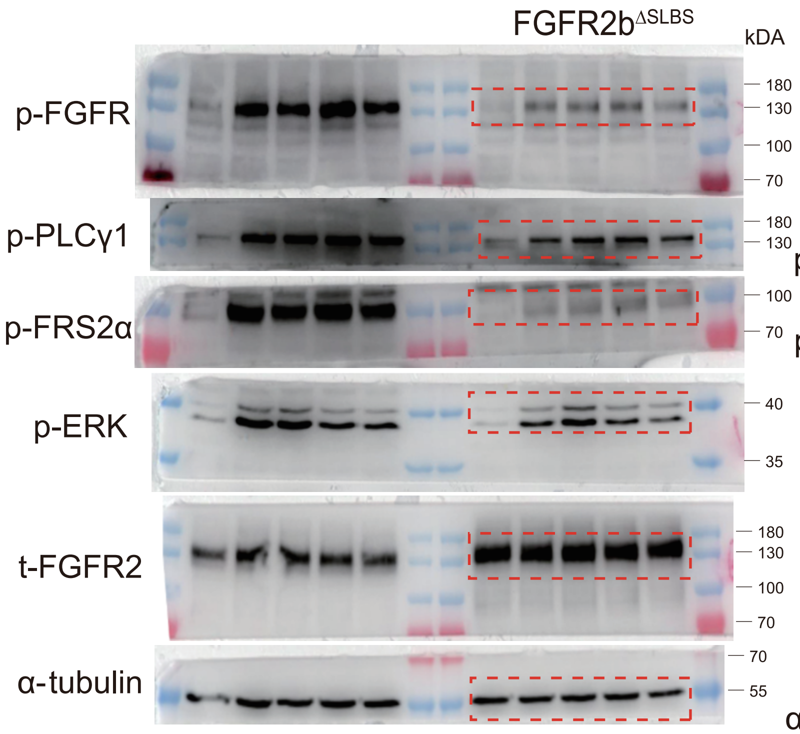

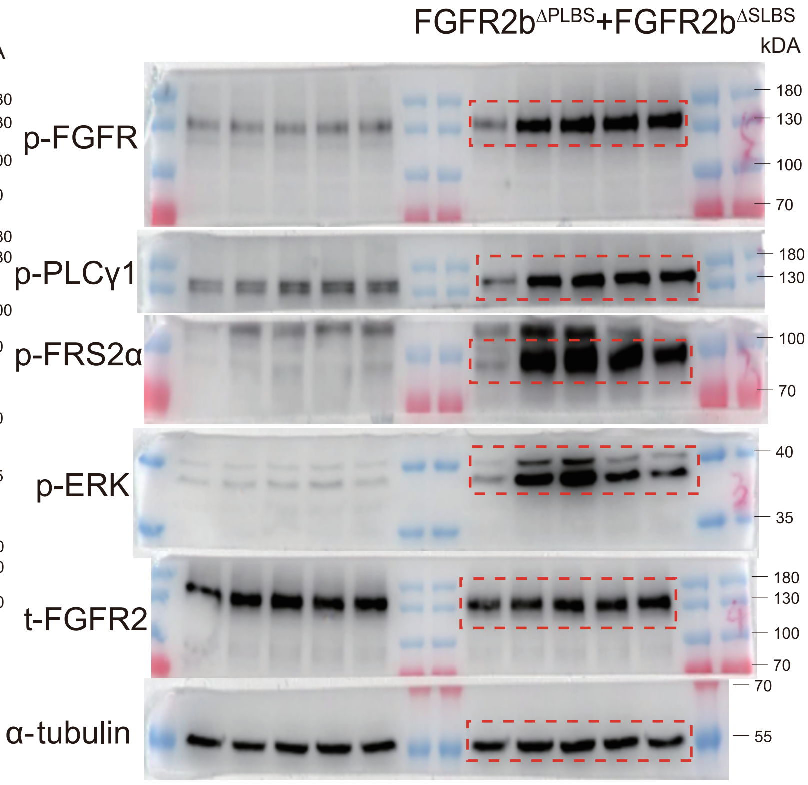

Uncropped Blotting For Extended Data Figure 7d

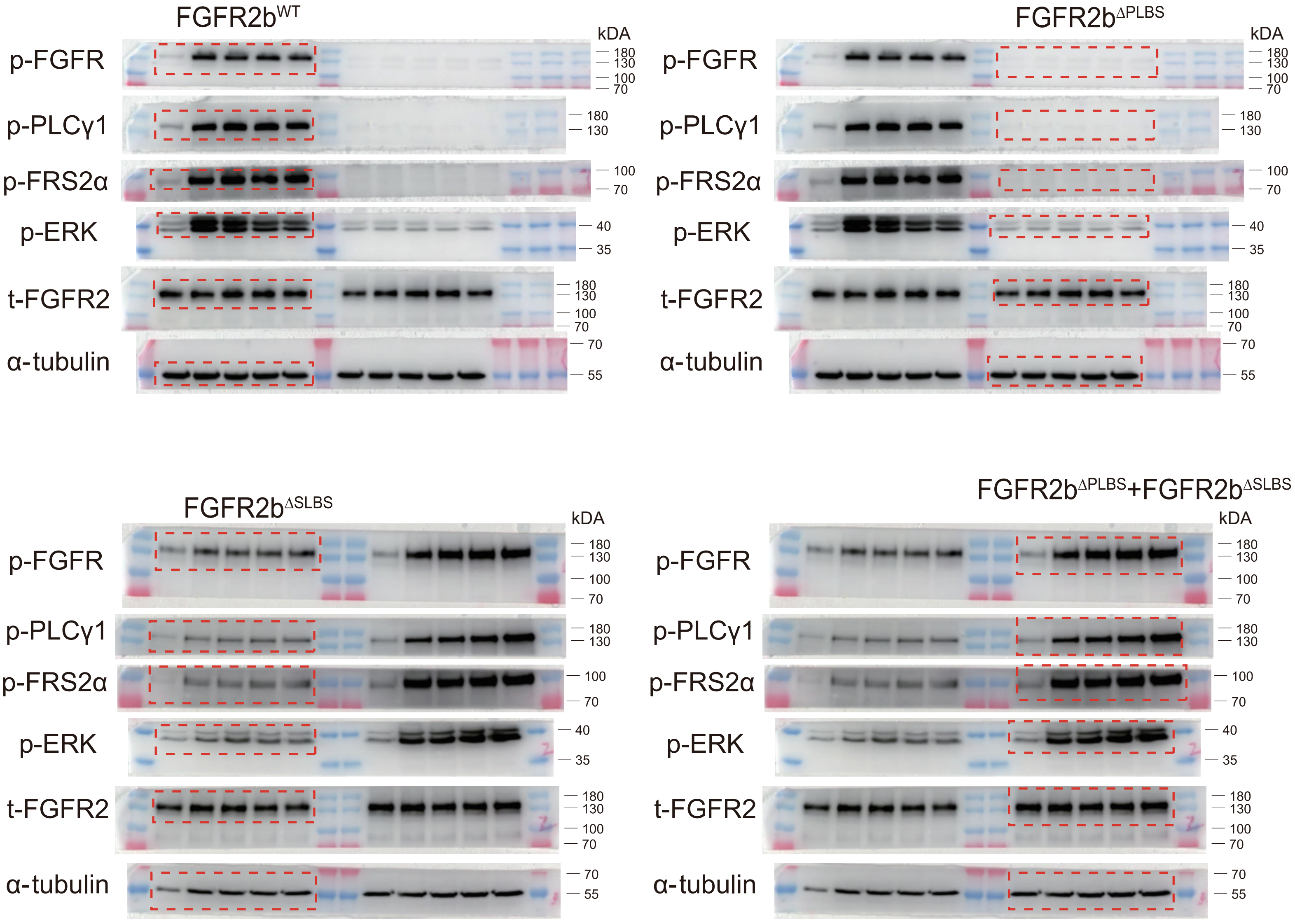

Uncropped Blotting For Extended Data Figure 7e

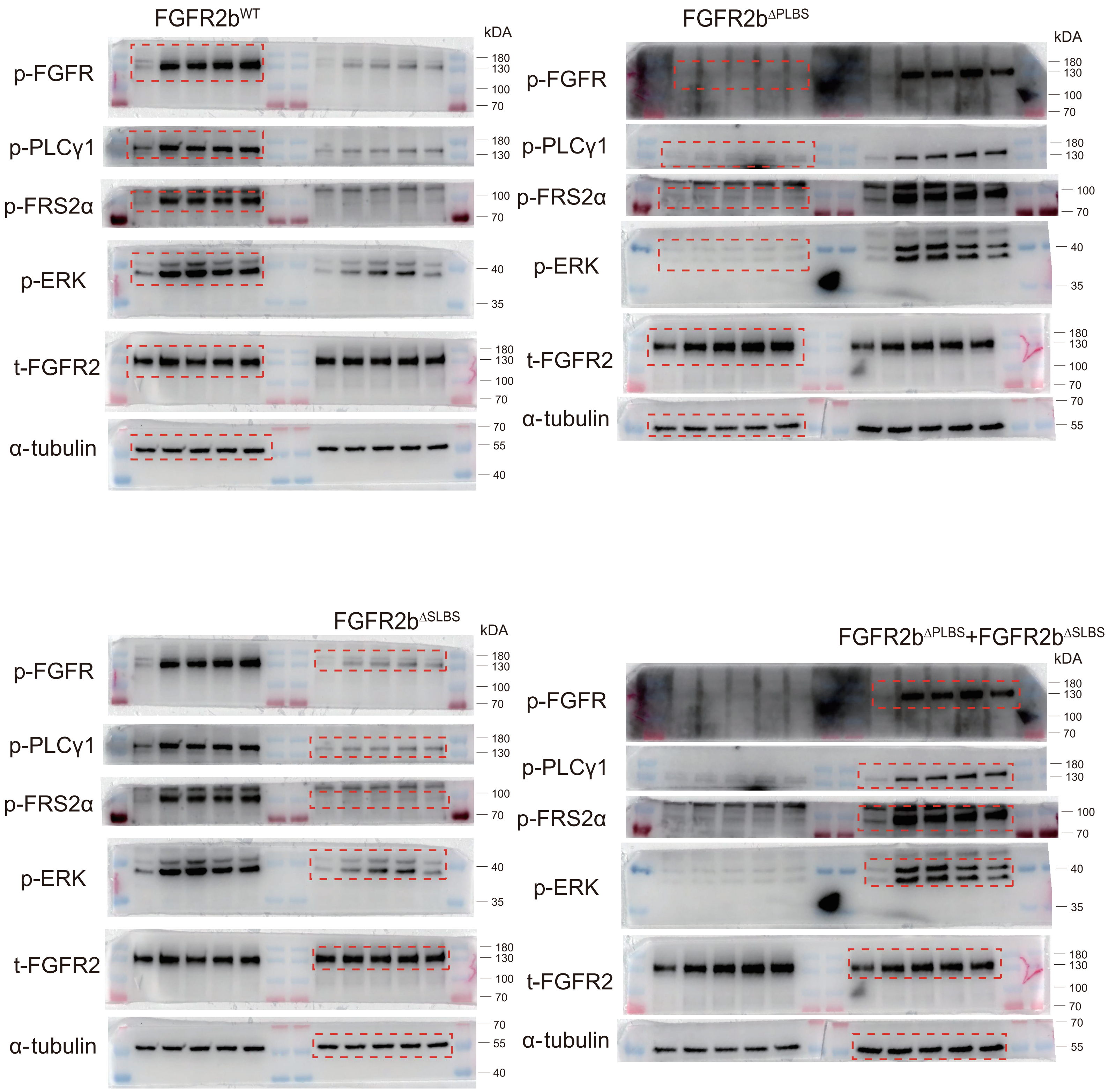

Uncropped Blotting For Extended Data Figure 8b

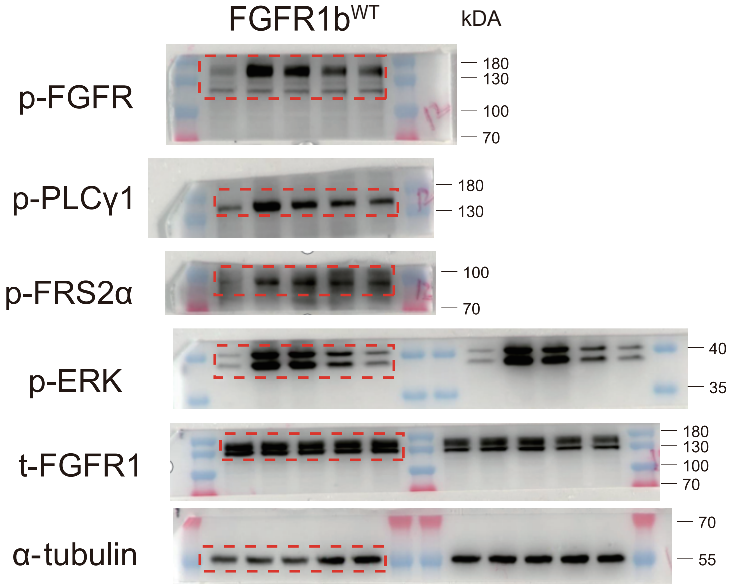

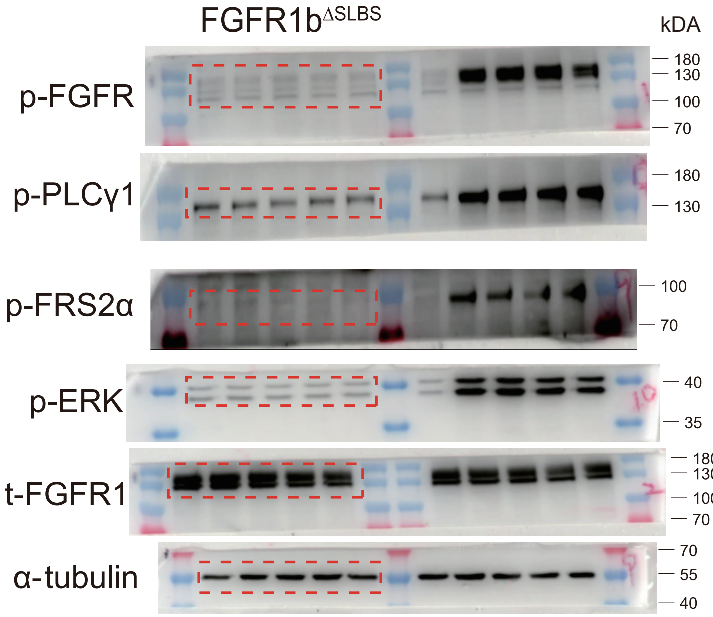

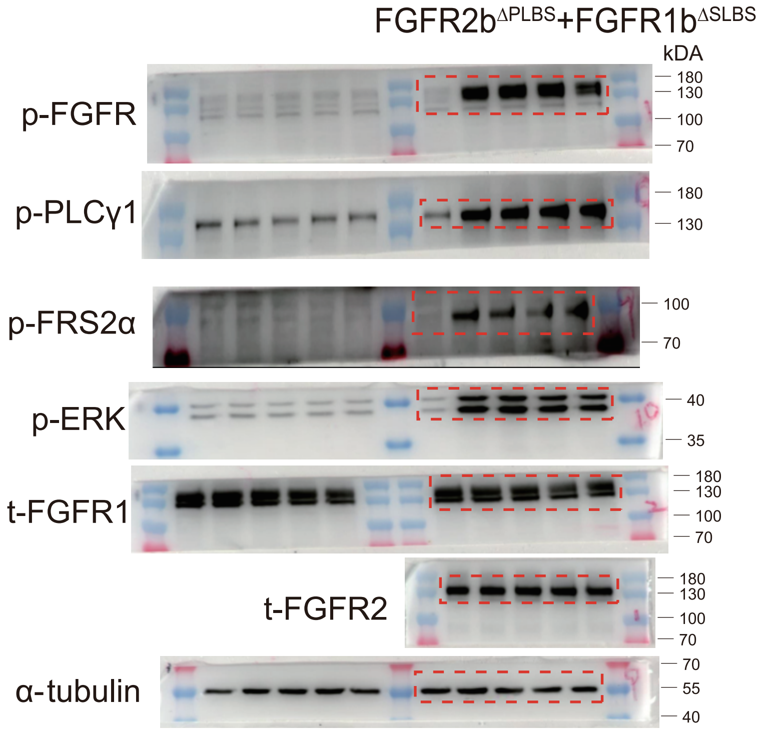

Uncropped Blotting For Extended Data Figure 8c

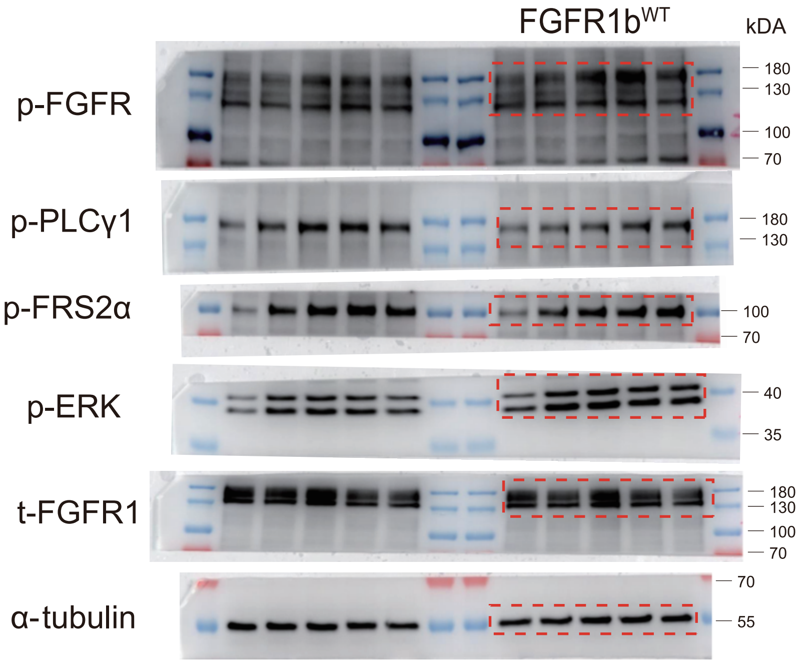

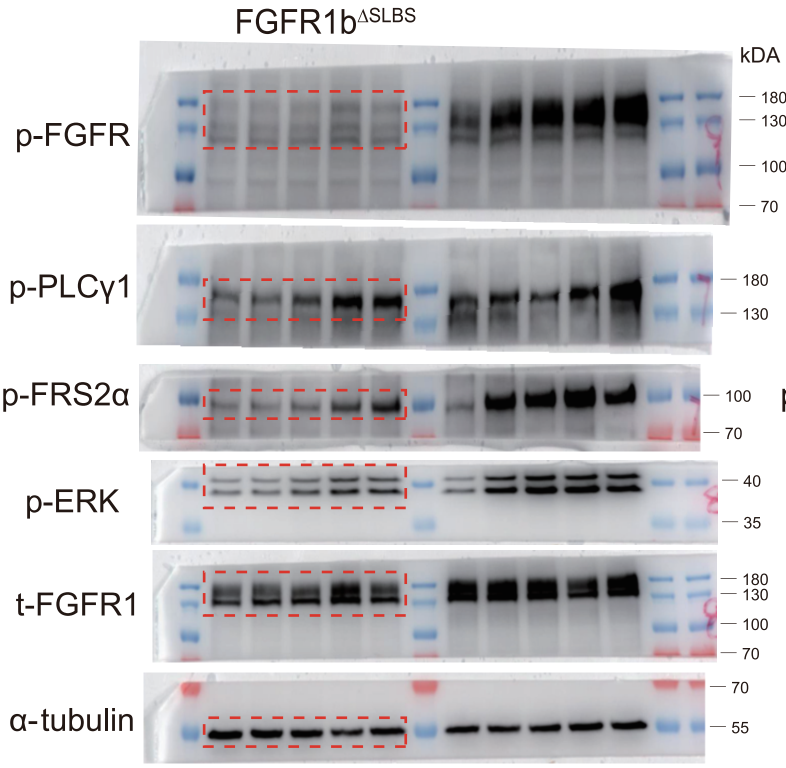

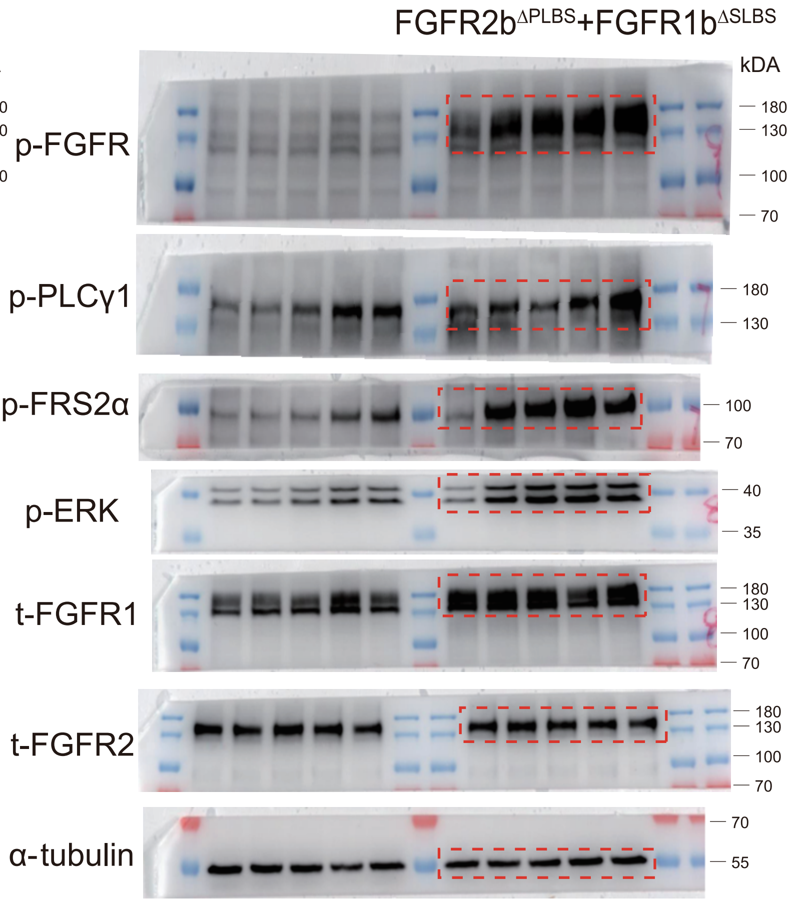

Uncropped Blotting For Extended Data Figure 8e

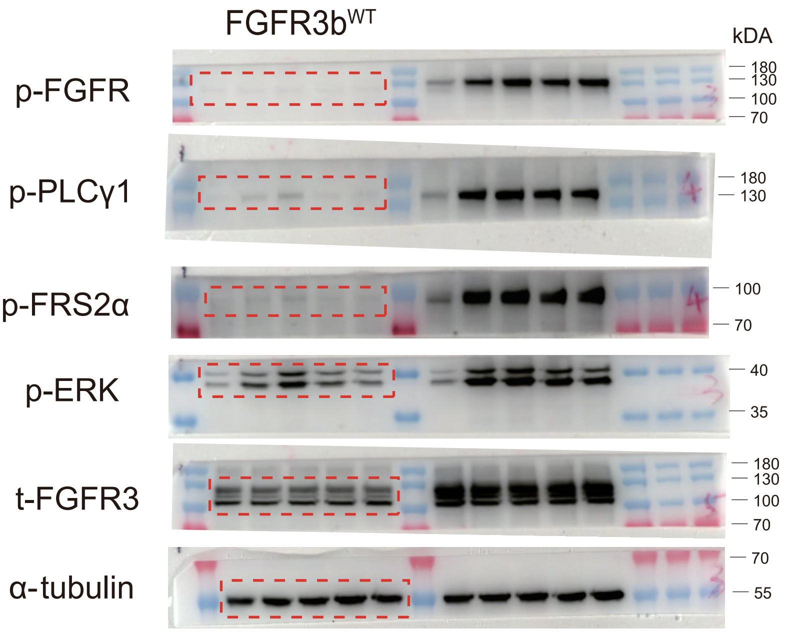

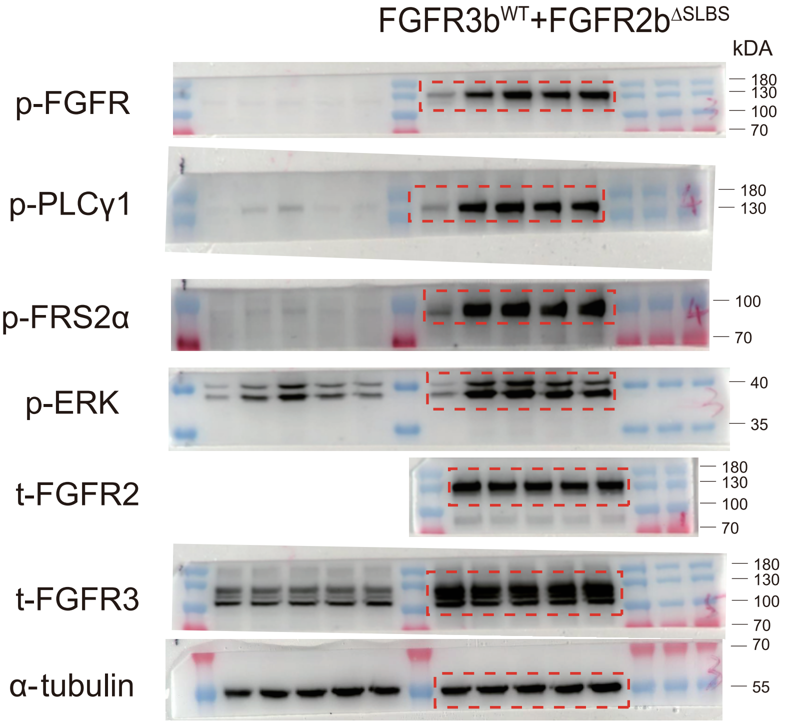

Uncropped Blotting For Supplementary Figure3

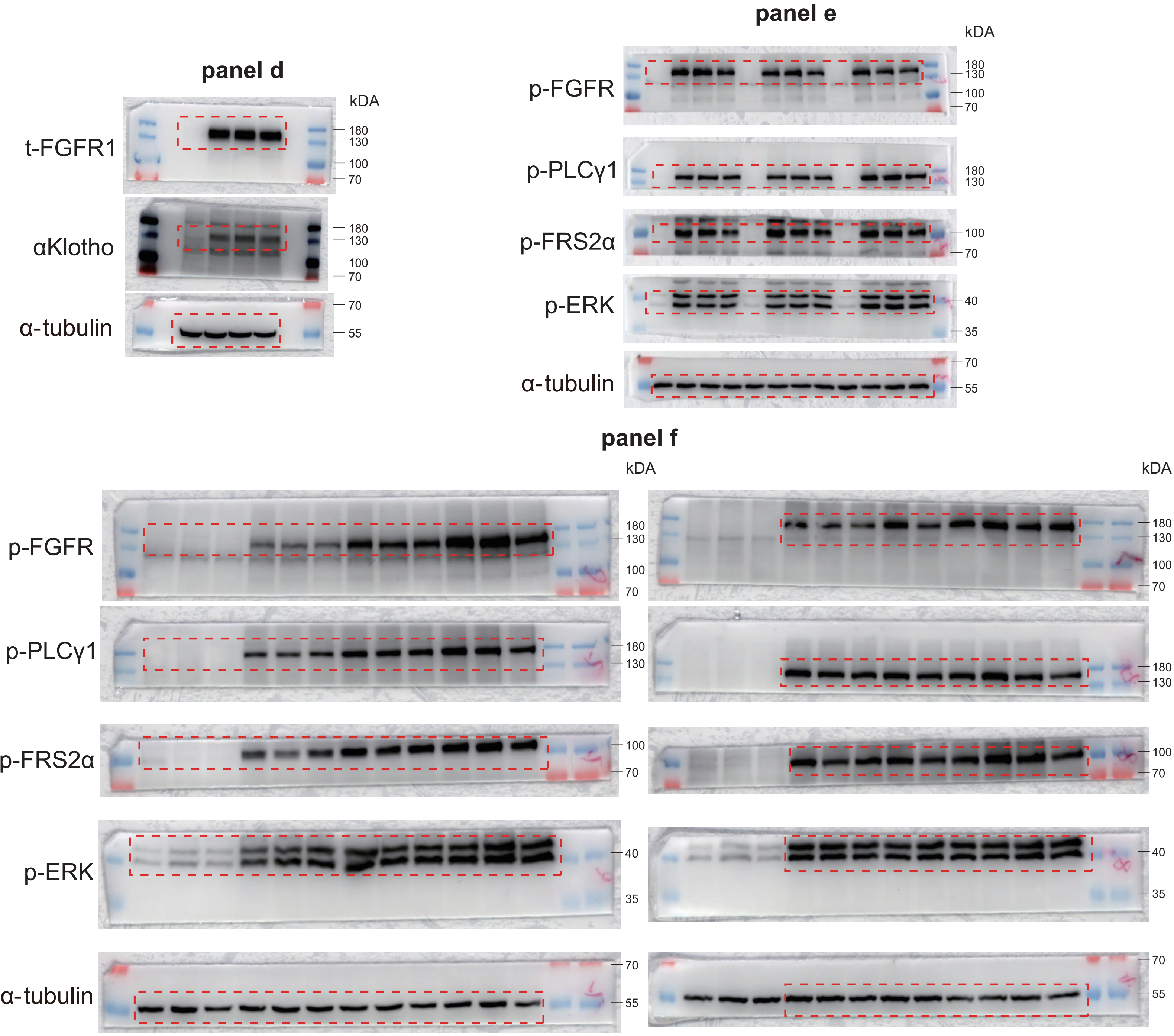

Supplementary Fig.2. Purification of all three physiologically possible human FGF23-FGFR-αKlotho-HS quaternary complexes.

Representative size exclusion chromatograms of FGF23-FGFR1c-αKlotho-HS (**a**), FGF23-FGFR3c-αKlotho-HS (**b**), and FGF23-FGFR4-αKlotho-HS (**c**) quaternary complexes obtained over Superdex 200 column. Under the chromatograms, Coomassie blue-stained SDS–PAGE gels show the contents of the indicated main peak fractions within the dashed lines. Results are from at least three replicates. Molecular weight markers are labelled. Bands corresponding to FGF23, FGFRs and αKlotho are indicated. **d,** Purified samples of $FGF23^{WT}$ and its mutants ($FGF23^{\Delta HBS}$, $FGF23^{\Delta NT}$, and $FGF23^{\Delta SRBS}$) were analyzed by SDS-PAGE confirming similar purity and quantity. **e,** Size exclusion chromatography-multi-angle light scattering (SEC–MALS) analysis of 1:1:1 mixture of FGF23, FGFR1c and αKlotho in the absence (green line) or presence of HS (blue line). Horizontal dashed line indicates molecular mass of the main peak.

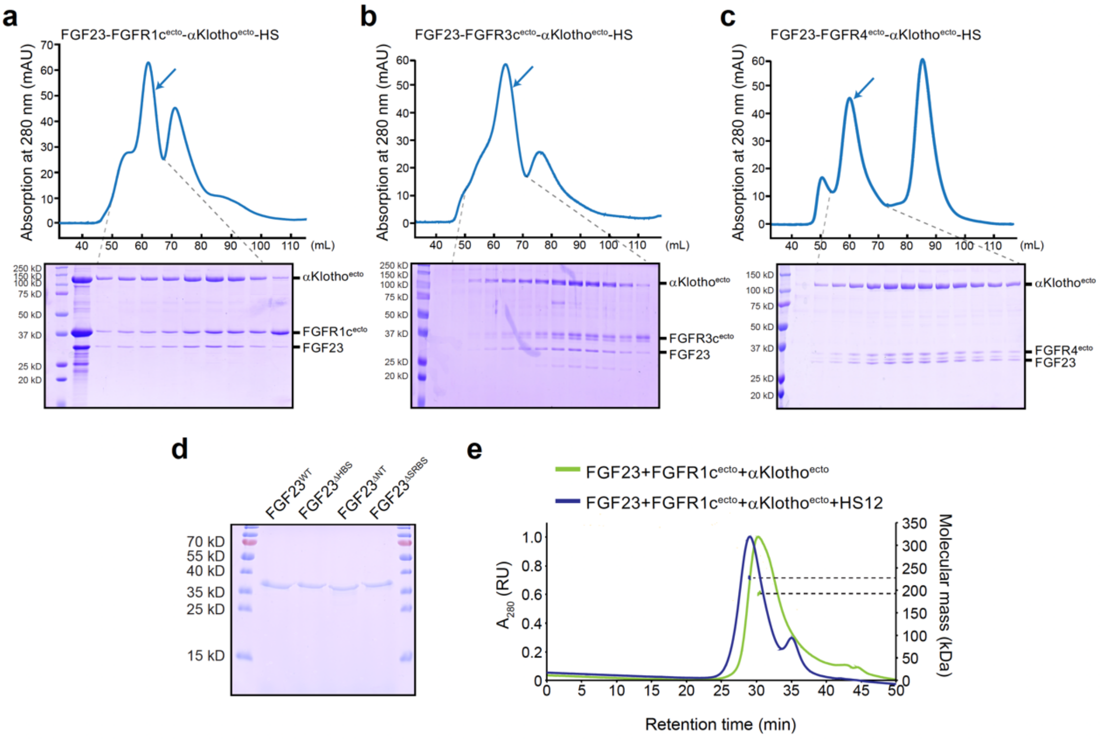

Supplementary Fig.3. Hypothetical 1:2:2:1 or 2:4:2:2 FGF23-FGFR-αKlotho-HS quaternary complexes.

a, Cryo-EM maps of FGF23-FGFR1c-αKlotho-HS (left), FGF23-FGFR3c-αKlotho-HS (center) and FGF23-FGFR4-αKlotho-HS (right) quaternary complexes shown at low threshold levels of 0.4, 0.3 and 0.3, respectively. Note the presence of an extra weak density (in black dashed box) adjacent to the 1:2:1:1 quaternary complex. **b**, Top: Surface rendering in two views showing that the extra density represents an additional αKlotho molecule ($\alpha Klotho^S$ in wheat color) that packs via its KL2 domain against the KL2 domain of αKlotho component ($\alpha Klotho^P$) of 1:2:1:1 quaternary complexes (colored as in Fig. 2a). Beneath the surface representation, the 1:2:1:1 FGF23-FGFR-αKlotho-HS quaternary complex and $\alpha Klotho^S$ are pulled apart and rotated along the vertical axis to expose the $\alpha Klotho^P$-$\alpha Klotho^S$ contact sites (in purple). Cartoon representation of the boxed region highlighting residues (rendered in purple sticks) at the αKlotho-αKlotho interface. **c**, Based on this observation, we contemplated the possibility that FGF23 signaling may entail higher order assemblies featuring a 1:2:2:1 (left) or a 2:4:2:2 stoichiometry (right), the latter resulting from symmetric apposition of two sets of 1:2:1:1 FGF23-FGFR1c-αKlotho-HS quaternary complexes. Such higher order complexes seemed plausible particularly on cell membrane where reduced dimensionality would promote weak interactions between transmembrane αKlotho molecules. **d-f,** FGF23 signaling does not require 1:2:2:1 or 2:4:2:2 FGF23-FGFR1c-αKlotho-HS complexes. Wild-type transmembrane αKlotho ($\alpha Klotho^{TM}$) or its mutated versions harboring either a N782H/F784S/Y788R or D776A/N779A/Q780S triple mutation - introduced to disrupt αKlotho-αKlotho contacts - were co-expressed with $FGFR1c^{WT}$ in L6 cell lines. Expression levels of FGFR1c and $\alpha Klotho^{TM}$ were confirmed by western blotting (**d**). These cell lines were exposed to a fixed concentration of FGF23 for increasing time intervals (**e**) or varying concentrations of FGF23 for a fixed time (**f**). FGFR1c activation/signaling was monitored by western blotting analysis of total cell lysates with antibodies against phosphorylated FGFR and its downstream signal transducers. Note that in both sets of experiments, αKlotho mutants elicited comparable capacity as wild type $\alpha Klotho^{TM}$ in promoting FGF23 signaling as evident by similar levels of FGFR1c/PLCγ1/FRS2α/ERK phosphorylation. These cell-based mutagenesis data strongly argued against a physiological role for higher order 1:2:2:1 or 2:4:2:2 FGF23-FGFR1c-αKlotho-HS assemblies in FGF23 signaling and accordingly, we did not consider them further. Experiments were performed in biological triplicates with similar results.

Continued

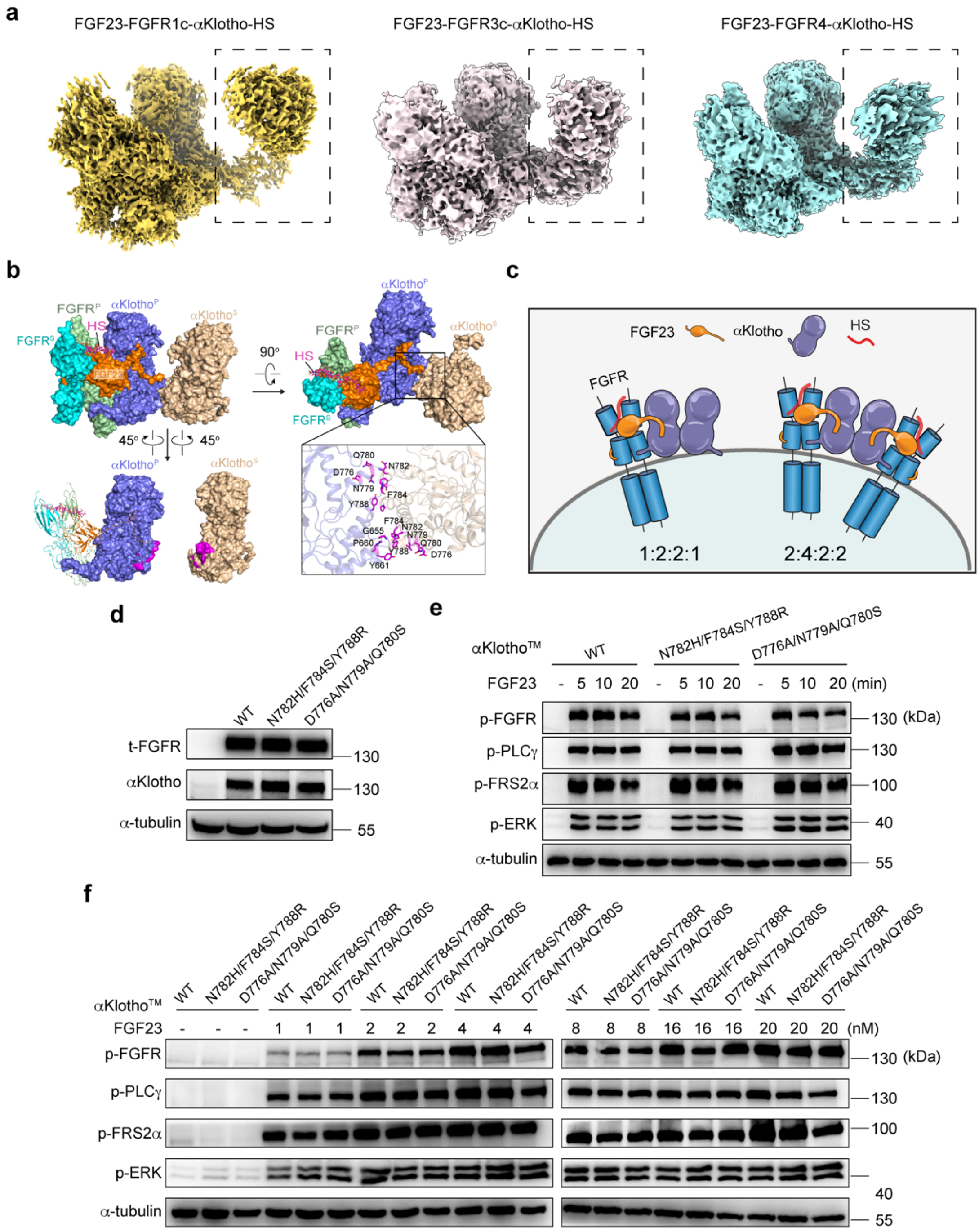

a
FGF23-FGFR1c-αKlotho-HS
FGF23-FGFR3c-αKlotho-HS
FGF23-FGFR4-αKlotho-HS
b
FGFR^P
αKlotho^P
αKlotho^S
FGFR^S
HS
FGF23
90°
45°
45°
Q780
N782
D776
N779
F784
Y788
G655
P660
Y661
c
FGF23
αKlotho
HS
FGFR
1:2:2:1
2:4:2:2
d
WT
N782H/F784S/Y788R
D776A/N779A/Q780S
t-FGFR
αKlotho
α-tubulin
130
130
55
e
αKlotho^TM
WT
N782H/F784S/Y788R
D776A/N779A/Q780S
FGF23
- 5 10 20 - 5 10 20 - 5 10 20 (min)
p-FGFR
p-PLCγ
p-FRS2α
p-ERK
α-tubulin
130 (kDa)
130
100
40
55
f
αKlotho^TM
FGF23
- - - 1 1 1 2 2 2 4 4 4 8 8 8 16 16 16 20 20 20 (nM)
p-FGFR
p-PLCγ
p-FRS2α
p-ERK
α-tubulin
130 (kDa)
130
100
40
55

Supplementary Fig.4. FGFR^P-FGFR^S contacts deprive FGFR^S from FGF23 binding by inducing a D3 rotation and by occluding ligand binding sites in FGFR^S.

a, Left: ribbon diagram of 1:2:1 FGF23-FGFR1c-HS component of the quaternary complex. αKlotho is omitted for simplicity. Center: superimposition of FGFR^S (cyan) onto FGFR^P (pale green) via D2 domain alignment reveals a large rotation of D3 domain of FGFR^S pivoted around C-terminal pole of D2 domain. This rotation is caused by contacts with FGFR^P and FGF23 and renders the conformation of FGFR^S incompatible with FGF23 binding. Right: zoomed-in view of the boxed region showing that if another FGF23 were to bind FGFR^S, its core region would sterically clash with the FGFR^S D3 domain. FGFR^P is omitted for clarity. **b**, Center: Cartoon representation of the quaternary complex with boxes demarcating the contacts involving D2-D3 linker and top (N-terminal) end of D3 domain of FGFR^P and FGFR^S chains. Left: expanded view of the boxed region showing that the D2-D3 linker and the βF-βG loop in D3 of FGFR^P play critical roles in ligand binding. Specifically, Arg-250 from the D2-D3 linker and Ser-346 from the βF-βG loop make hydrogen bonds with Asp-125 and His-117 of FGF23, respectively. Right: expanded view of the boxed region showing that the D2-D3 linker and βF-βG loop of FGFR1^S are trapped in the FGFR^P-FGFR^S dimer interface and hence are inaccessible for FGF23.

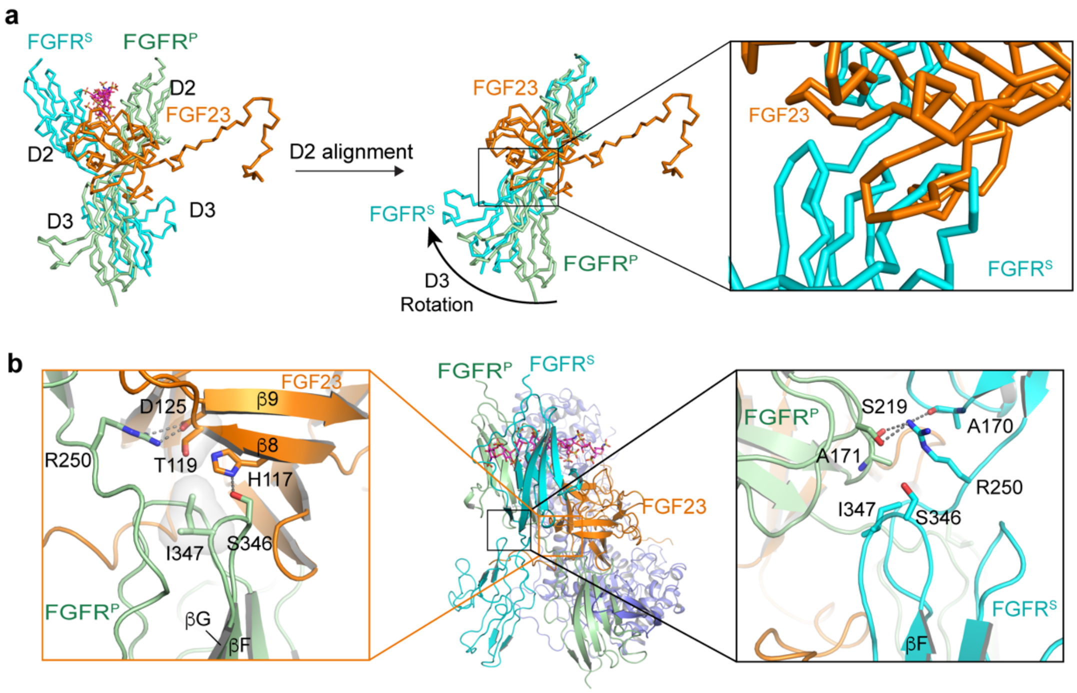

Supplementary Table 1. List of contacts at sites 1 and 2 of the asymmetric $FGFR^{P}$-$FGFR^{S}$ interface within the FGF23-FGFR1c-αKlotho-HS quaternary complex.

Contact sites	Residues of $FGFR^{P}$	Residues of $FGFR^{S}$
site 1 (hydrophobic interaction)	Ala-170, Ala-171, Val-221, Pro-222	Ile-347
site 1 (hydrogen bonds)	Ala-171, Ser-219	Arg-250
site 1 (hydrogen bonds)	Asp-218, Ser-219	Lys-172
site 2 (hydrophobic interaction)	Ile-256	Ile-256, Tyr-280
site 2 (hydrogen bonds)	Glu-249	Arg-254
site 2 (hydrogen bonds)	Arg-250	Pro-252
site 2 (hydrogen bonds)	Leu-257	Gln-258
site 2 (hydrogen bonds)	Ala-259	Ala-259

Supplementary Table 2. List of interactions at the FGF23-FGFR^{S} interface within the FGF23-FGFR1c-αKlotho-HS quaternary complex.

**Residues of FGF23**	**Residues of FGFR^{S}**
Pro-151, Leu-120 (core)	Ile-203
Glu-121 (core)	Ser-223
Asn-150 (core)	Asp-200, Arg-202
Try-25, Pro-26 (N-terminus)	Leu-290, Leu-342, Leu-349
Ser-29 (N-terminus)	Arg-254, Ser-350
Leu-31, and Leu-32 (N-terminus)	Ile-256, Ala-259
