# Supplementary material for: Structural basis for FGF hormone signalling
Source: Nature. 2023 Jun 7;618(7966):862–70. doi: 10.1038/s41586-023-06155-9 (PMC10284700; doi:10.1038/s41586-023-06155-9)
Supplement: Supplementary file 2 — Reporting Summary [file 41586_2023_6155_MOESM2_ESM.pdf]

nature portfolio

Corresponding author(s): Moosa Mohammadi

Last updated by author(s): Apr 5, 2023

Reporting Summary

Nature Portfolio wishes to improve the reproducibility of the work that we publish. This form provides structure for consistency and transparency in reporting. For further information on Nature Portfolio policies, see our Editorial Policies and the Editorial Policy Checklist.

Statistics

For all statistical analyses, confirm that the following items are present in the figure legend, table legend, main text, or Methods section.

n/a	Confirmed	
☐	☒	The exact sample size (*n*) for each experimental group/condition, given as a discrete number and unit of measurement
☐	☒	A statement on whether measurements were taken from distinct samples or whether the same sample was measured repeatedly
☐	☒	The statistical test(s) used AND whether they are one- or two-sided<br>*Only common tests should be described solely by name; describe more complex techniques in the Methods section.*
☒	☐	A description of all covariates tested
☒	☐	A description of any assumptions or corrections, such as tests of normality and adjustment for multiple comparisons
☐	☒	A full description of the statistical parameters including central tendency (e.g. means) or other basic estimates (e.g. regression coefficient) AND variation (e.g. standard deviation) or associated estimates of uncertainty (e.g. confidence intervals)
☐	☒	For null hypothesis testing, the test statistic (e.g. *F*, *t*, *r*) with confidence intervals, effect sizes, degrees of freedom and *P* value noted<br>*Give P values as exact values whenever suitable.*
☒	☐	For Bayesian analysis, information on the choice of priors and Markov chain Monte Carlo settings
☒	☐	For hierarchical and complex designs, identification of the appropriate level for tests and full reporting of outcomes
☒	☐	Estimates of effect sizes (e.g. Cohen's *d*, Pearson's *r*), indicating how they were calculated

Our web collection on statistics for biologists contains articles on many of the points above.

Software and code

Policy information about availability of computer code

Data collection	Leginon (version 3.5)<br>Data of Sec-Mals was collected by ASTRA 5.3.4.20<br>Grey values of band in Western blots were collected by ImageJ 1.57j8
Data analysis	CryoSparc (version 2.15); Phenix suite (version 1.9_1692); COOT (version 0.8.2); PyMOL (version 2.5.2); UNICORN (version 7.6, GE Healthcare, USA); UCSF Chimera X (version 1.3); GraphPad (version 8.0, GraphPad Software, Inc., USA).

For manuscripts utilizing custom algorithms or software that are central to the research but not yet described in published literature, software must be made available to editors and reviewers. We strongly encourage code deposition in a community repository (e.g. GitHub). See the Nature Portfolio guidelines for submitting code & software for further information.

Data

Policy information about availability of data

All manuscripts must include a data availability statement. This statement should provide the following information, where applicable:

- Accession codes, unique identifiers, or web links for publicly available datasets
- A description of any restrictions on data availability
- For clinical datasets or third party data, please ensure that the statement adheres to our policy

Electron density maps and refined models for the FGF23–FGFR1c–aKlotho–HS (EMD-34075, 7YSH), FGF23–FGFR3c–aKlotho–HS (EMD-34082, 7YSU) and FGF23–

FGFR4–aKlotho–HS (EMD-34084, 7YSW) quaternary complexes have been deposited in the Electron Microscopy Data Bank and will be released upon acceptance of the manuscript. All the data are available within the paper and its supplementary information files. Source data are provided with this paper.

Human research participants

Policy information about studies involving human research participants and Sex and Gender in Research.

Reporting on sex and gender	n/a
Population characteristics	n/a
Recruitment	n/a
Ethics oversight	n/a

Note that full information on the approval of the study protocol must also be provided in the manuscript.

Field-specific reporting

Please select the one below that is the best fit for your research. If you are not sure, read the appropriate sections before making your selection.

☒ Life sciences ☐ Behavioural & social sciences ☐ Ecological, evolutionary & environmental sciences

For a reference copy of the document with all sections, see nature.com/documents/nr-reporting-summary-flat.pdf

Life sciences study design

All studies must disclose on these points even when the disclosure is negative.

Sample size	Sample sizes are given in the manuscript. All the cell based FGFR autophosphorylation and signaling studies were repeated at least three times and PLA data were generated by six randomly chosen microscope fields from at least two independent experiment, which is sufficient to derive error bars, p values and statistical significance. No sample-size calculation was performed.
Data exclusions	No data were excluded from the analyses.
Replication	All replication were successful, and the replication numbers are either mentioned in the figure legends or Methods section.<br>Protein purifications were repeated at least 8 times and showed similar chromatography and electrophoresis patterns.<br>Western blot experiments were repeated in biological triplicates.<br>PLA assay were repeated at least two times independently.
Randomization	PLA data were generated by six randomly chosen microscope fields from at least two independent experiment. For all other experiments, all data were used for analysis, thus no randomization was needed.
Blinding	Blinding applied during protein complex particle picking and Ab-initio reconstruction and heterogeneous refinement. Other experiments does not relevant to blinding as no groups were assigned.

Reporting for specific materials, systems and methods

We require information from authors about some types of materials, experimental systems and methods used in many studies. Here, indicate whether each material, system or method listed is relevant to your study. If you are not sure if a list item applies to your research, read the appropriate section before selecting a response.

Materials & experimental systems

n/a	Involved in the study	
☐	☒	Antibodies
☐	☒	Eukaryotic cell lines
☒	☐	Palaeontology and archaeology
☒	☐	Animals and other organisms
☒	☐	Clinical data
☒	☐	Dual use research of concern

Methods

n/a	Involved in the study	
☒	☐	ChIP-seq
☒	☐	Flow cytometry
☒	☐	MRI-based neuroimaging

Antibodies

Antibodies used

phosphorylated FGFR (#3471S, Cell Signaling Technology, USA); phosphorylated FRS2α (#3864S, Cell Signaling Technology, USA); phosphorylated PLCγ1 (#2821S, Cell Signaling Technology, USA); phosphorylated ERK1/2 (#4370S, Cell Signaling Technology, USA); α-tubulin (#66031-1-Ig, Proteintech, China); total-FGFR1 (#9740S, Cell Signaling Technology, USA), total-FGFR2 (#23328S, Cell Signaling Technology, USA), total-FGFR3 (#ab133644, Abcam, UK), total-FGFR4 (#8562S, Cell Signaling Technology, USA), HRP conjugated Goat anti-mouse IgG (H+L) (#SA00001-1, Proteintech, China), HRP conjugated Goat Anti-Rabbit IgG(H+L) (#SA00001-2, Proteintech, China); FGFR1 (#PA5-25979, ThermoFisher, USA); FGFR1 (#ab824, Abcam, UK); FGFR4 (#sc-136988, Santa Cruz Biotechnology, USA)

Validation

All of the antibodies used for western experiments and PLA are commercially available products, validation are available on manufacturers' websites.
anti-phosphorylated FGFR, Cell Signaling Technology, 3471S, western blot (1:1000): https://www.cellsignal.cn/products/primary-antibodies/phospho-fgf-receptor-tyr653-654-antibody/3471
anti-phosphorylated FRS2α, Cell Signaling Technology, 3864S, western blot (1:1000): https://www.cellsignal.cn/products/primary-antibodies/phospho-frs2-a-tyr196-antibody/3864
anti-phosphorylated PLCγ1, Cell Signaling Technology, 2821S, western blot (1:1000): https://www.cellsignal.cn/products/primary-antibodies/phospho-plcg1-tyr783-antibody/2821
anti-phosphorylated ERK1/2, Cell Signaling Technology, 4370S, western blot (1:1000): https://www.cellsignal.cn/products/primary-antibodies/phospho-p44-42-mapk-erk1-2-thr202-tyr204-d13-14-4e-xp-rabbit-mab/4370
anti-α-tubulin, Proteintech, 66031-1-Ig, western blot (1:20000): https://ptgcn.com/products/tubulin-Alpha-Antibody-66031-1-Ig.htm
anti-total-FGFR1, Cell Signaling Technology, 9740S, western blot (1:1000): https://www.cellsignal.cn/products/primary-antibodies/fgf-receptor-1-d8e4-xp-rabbit-mab/9740
anti-total-FGFR2, Cell Signaling Technology, 23328S, western blot (1:1000): https://www.cellsignal.cn/products/primary-antibodies/fgf-receptor-2-d4l2v-rabbit-mab/23328
anti-total-FGFR3, Abcam, ab133644, western blot (1:1000): https://www.abcam.cn/products/primary-antibodies/fgfr3-antibody-epr23043-ab133644.html
anti-total-FGFR4, Cell Signaling Technology, 8562S, western blot (1:1000) and PLA (1:100): https://www.cellsignal.cn/products/primary-antibodies/fgf-receptor-4-d3b12-xp-rabbit-mab/8562
anti-HRP conjugated Goat anti-mouse IgG (H+L), Proteintech, SA00001-1, western blot (1:5000): https://ptgcn.com/products/HRP-conjugated-Affinipure-Goat-Anti-Mouse-IgG-H-L-secondary-antibody.htm
anti-HRP conjugated Goat anti- Rabbit IgG (H+L), Proteintech, SA00001-2, western blot (1:5000): https://ptgcn.com/products/HRP-conjugated-Affinipure-Goat-Anti-Rabbit-IgG-H-L-secondary-antibody.htm
anti-FGFR1, ThermoFisher, PA5-25979, PLA (1:20): https://www.thermofisher.cn/cn/zh/antibody/product/FGFR1-Antibody-Polyclonal/PA5-25979
anti-FGFR1, Abcam, ab824, PLA (1:100): https://www.abcam.cn/products/primary-antibodies/fgfr1-antibody-m5g10-ab824.html
anti-FGFR4, Santa Cruz Biotechnology, sc-136988, PLA (1:100): https://www.scbt.com/zh/p/fgfr-4-antibody-a-10

Eukaryotic cell lines

Policy information about cell lines and Sex and Gender in Research

Cell line source(s)

L6 myoblast cell line (#GNR 4, National Collection of Authenticated Cell Cultures, China); HEK293T cells (kindly provided by Cell Bank/Stem Cell Bank, Chinese Academy of Sciences, China); N-acetylglucosaminytransferase I (GnTI) deficient HEK293S cells (#CRL-3022, American Type Culture Collection, USA).

Authentication

The cell lines were identified by morphology check under microscope in the lab.

Mycoplasma contamination

Mycoplasma negative per DAPI staining.

Commonly misidentified lines
(See ICLAC register)

No commonly misidentified cell lines were used.
